# Effect of Repeated Home Quarantine on Anxiety, Depression, and PTSD Symptoms in a Chinese Population During the COVID-19 Pandemic: A Cross-sectional Study

**DOI:** 10.3389/fpsyt.2022.830334

**Published:** 2022-05-16

**Authors:** Qiao Tang, Ya Wang, Jing Li, Dan Luo, Xiaoting Hao, Jiajun Xu

**Affiliations:** ^1^Department of Psychiatry and Mental Health, West China Hospital and West China School of Clinical Medicine, Sichuan University, Chengdu, China; ^2^Department of Nursing, West China Hospital and West China School of Nursing, Sichuan University, Chengdu, China; ^3^Department of Neurology, West China Hospital and West China School of Clinical Medicine, Sichuan University, Chengdu, China

**Keywords:** COVID-19, quarantine, anxiety, depression, post-traumatic stress disorder (PTSD)

## Abstract

**Background:**

Strict quarantines can prevent the spread of the COVID-19 pandemic, but also increase the risk of mental illness. This study examined whether the people who have experienced repeated home quarantine performance more negative emotions such as anxiety, depression, and post-traumatic stress disorder (PTSD) in a Chinese population.

**Methods:**

We collected data from 2,514 participants in Pi County, Chengdu City, and stratified them into two groups. Group 1 comprised 1,214 individuals who were quarantined only once in early 2020, while Group 2 comprised 1,300 individuals who were quarantined in early 2020 and again in late 2020. Both groups were from the same community. The GAD-7, PHQ-9, and PCL-C scales were used to assess symptoms of anxiety, depression, and PTSD between the two groups.

**Results:**

Analyses showed that total PHQ-9 scores were significantly higher in Group 2 than in Group 1 (*p* < 0.001) and the quarantine times and age are independent predictors of symptoms of depression (*p* < 0.001). The two groups did not differ significantly in total GAD-7 or PCL-C scores.

**Conclusion:**

Increasing quarantine times was associated with moderate to severe depression symptoms, but not with an increase in symptoms of anxiety or PTSD.

## Introduction

As the Black Swan event of the 21^st^ century, the COVID-19 epidemic has had a huge impact on us. A study of 10 countries with the highest number of reported deaths, suggests that the total number of infections is expected to reach 16 millions by July 2020 (1). Despite epidemic prevention measures such as quarantines, the global situation remains uncertain (2, 3). The COVID-19 pandemic poses a serious threat to the physical health and livelihood of people across the world, and it continues to have a considerable impact on their mental health (4). Over the last year, psychiatrists have reported a dramatic increase in the number of patients with anxiety, depression, and post-traumatic stress disorder (PTSD) (5). A study involving Chinese people reported that 53.8% suffered moderate or severe psychological impacts during the early stages of the pandemic (4). Another survey reported that the prevalence of anxiety among Chinese university and college students has increased from 24.9 to 31% during the pandemic, while the prevalence of depression has increased from 9 to 41.8% (6).

Apart from the panic and stress caused by the COVID-19 outbreak itself, home quarantine has had a significant effect on public mental health. Quarantine is considered an effective measure for the prevention and control of epidemics, since they restrict the movement of people and therefore can slow the spread of the disease. Nevertheless, quarantine can cause several issues, including an increase in psychological burden due to reduced contact with the outside world (7), as well as an increase in mental health disorders due to reduced physical activity and increased economic pressure (8). During quarantine, individuals may be more likely to experience negative emotional and behavioral problems, such as despair, anxiety, guilt, insomnia, anger, fear, alcoholism, and smoking (9–11). Social isolation and the lack of interpersonal communication can also lead to, or aggravate, depression (12).

In early 2020, at the start of the COVID-19 pandemic, Chinese people had carried out 14-days quarantine between February and March. Despite the considerable efforts being made in China to fight the spread of COVID-19 (13), sporadic, small-scale outbreaks have occurred in different parts of the country. For example, the COVID-19 outbreak in Pi County, Chengdu City had a strong impact on the local population, and much of the population was quarantined a second time in late 2020, for periods ranging from December 8, 2020 to December 22, 2020. In this study, we investigated the psychological impact of repeated home quarantine on the Chinese population in Pi County during the COVID-19 pandemic. We are not aware of other studies addressing the effect of repeated quarantine on the risk of anxiety, depression, or PTSD. The findings of the present study may help improve interventions to counteract negative effects of quarantine, such as during epidemics.

## Methods

### Study Subjects

This cross-sectional case control study was conducted between April 2020 and April 2021. We recruited individuals from Pi County who had previously experienced home quarantine, and stratified them into two groups: those who had been quarantined 14 days only once between February and March (Group 1), and those who had been quarantined both in early and late 2020 (Group 2). Each quarantine period lasted 14 days. Both groups were from the same community and living environment and other basic circumstances were roughly similar.

We included only those who hadn't been diagnosed with SARS-CoV-2 infection previously and were at least 18 years old and under 65 years old in December 2019. We excluded participants with a history of anxiety, depression, PTSD, or other uncontrolled psychosis that had been diagnosed before quarantine, as well as those who were unable to read or understand the questionnaire.

This survey-based study was approved by the Ethics Committee of Sichuan University (Approval No.K2020006), and participants gave informed consent.

### Questonnaire Survey

Between 31 April 2020 and 01 March 2021, we conducted a cross-sectional network survey to collect data on symptoms of depression, anxiety, and PTSD experienced by the participants during quarantine. The study was performed based on the strategy of online sampling survey, and a custom-designed questionnaire was distributed to participants *via* WeChat.

Apart from relevant data on psychological status, we collected information on age, sex, number of years of education, marital status, and time spent in quarantine. Previous studies have shown that age (12) and level of education (13) can affect the psychological and emotional status of individuals affected by the pandemic. Therefore, for further analysis, we stratified participants based on age (<30 or ≥30 years) or number of years of education (<12 or > 12).

### Psychological Assessments

In the present study, these three scales were used to assess negative emotions and evaluate whether the two groups of participants differed significantly in symptoms related to anxiety, depression, or PTSD. The higher the scores on each scale, the more serious the symptoms.

The General Anxiety Disorder Scale-7 (GAD-7), the Patient Health Questionnaire (PHQ-9), and the PTSD Checklist-Civilian version (PCL-C) are widely used to evaluate anxiety, depression, and PTSD.

For the purpose of our study, the anxiety assessment scale was translated into Chinese and revised based on the GAD-7 scale before being incorporated into the questionnaire. The GAD-7 scale consists of seven items, and anxiety is scored as follows: minimum, 0–4; mild, 5–9; moderate, 10–14; and severe 15–21 (14).

The PHQ-9 questionnaire is typically used to screen depression in primary health care and other medical settings, and was developed based on nine criteria outlined in the Fourth Edition of the Diagnostic and Statistical Manual of Mental Disorders (DSM). Respondents answer items on a four-point Likert scale from 0 (“not at all”) to 3 (“almost every day”). Based on the total score, the severity of depression symptoms is categorized as follows: few symptoms of depression, 0–4; have symptoms of depression, >4 (15).

The PCL-C scale was developed according to the DSM-IV. This scale is based on 17 items, for which responses can range from 1 (“not at all”) to 5 (“extreme”). Based on the total score, patients are categorized as showing no obvious PTSD symptoms, 17–37; PTSD symptoms present to a certain extent, 38–49; and obvious symptoms that could lead to a diagnosis of PTSD (16).

### Statistical Analysis

Statistical analyses were performed using SPSS 23.0 (IBM, Chicago, IL, USA). We tested the effects of demographic factors such as age, sex, number of years of education, marital status, and exposure history on the psychological status of participants. Inter-group differences were assessed for significance using Student's *t*-tests and chi-squared tests. Multivariate logistic regression was used to independently analyze the factors associated with anxiety, depression, and PTSD. The multivariate model comprised variables that had a significant association with psychological status (*p* < 0.1) in the univariate analysis. All tests were bilateral, and the significance level was set at 0.01.

## Results

Of 2,651 questionnaires submitted, 137 were excluded because participants did not meet the age criteria, reported a history of mental illness, or failed to complete all items on the questionnaires. The remaining 2,514 questionnaires were retained in the final analysis and were submitted by individuals who had been quarantined at home in 2020.

### Demographic Characteristics

Group 1 comprised 1,214 individuals who had experienced only one home quarantine, while Group 2 comprised 1,300 individuals who had experienced home quarantine twice. While the first 14-days quarantine between February and March took place nationwide, the second quarantine ranging from December 8, 2020 to December 22, 2020 took place only in Pi County. A large proportion of participants in Group 2 were ≥30 years old (87.8%), higher percentage of people with <12 years of education (51.5%) and married (79.3%; Table 1), reflecting the remote, rural location of Pi County.

**Table 1:** Demographic characteristics of participants stratified based on quarantine times.

**Characteristic**	**Group 1** **(*n* = 1,214)**	**Group 2** **(*n* = 1,300)**	***p***
**Sex**			0.433
Male	441 (36.3)	492 (37.8)	
**Age (years)**			<0.01
<30	358 (29.5)	159 (12.2)	
≥30	856 (70.5)	1,141 (87.8)	
**Length of education (years)**			<0.01
<12	274 (22.6)	670 (51.5)	
>12	940 (77.4)	630 (48.5)	
**Marital status**			<0.01
Married	777 (64)	1,031 (79.3)	
**POSD**			0.114
PHQ >4	415 (34.2)	484 (37.2)	
PHQ ≤ 4	799 (65.8)	816 (62.8)	

*POSD: the proportion of people in both groups who indicated symptoms of depression, defined as PHQ-9 score > 4 Values are n (%)*.

### Assessment of Psychological Status

Based on GAD-7 and PCL-C scores, independent-samples *t*-tests showed no significant differences between the two groups in symptoms of anxiety (Group 1, 3.58 ± 4.1; Group 2, 3.78 ± 5.07, *p* = 0.117) or PTSD (Group 1, 24.42 ± 8.86; Group 2, 25.25 ± 10.86, *p* = 0.035; Table 2). In contrast, Group 2 showed a significantly higher total PHQ-9 score than Group 1 (4.8 ± 6.12 vs. 3.96 ± 4.88; p < 0.001; Table 2).

**Table 2:** Symptoms of anxiety, depression, and PTSD in participants stratified by the quarantine times.

**Scale**	**Group 1**	**Group 2**	***t***	***p***
GAD-7	3.58 ± 4.1	3.87 ± 5.07	1.57	0.117
PHQ-9	3.96 ± 4.88	4.8 ± 6.12	3.82	**<**0.001
PCL-C	24.42 ± 8.86	25.25 ± 10.86	2.11	0.035

*GAD-7, Generalized Anxiety Disorder-7; PCL-C, The PTSD Cheeklist-Civilian Version; PHQ-9, Patient Health Questionnaire-9*.

### Factors Associated With Symptoms of Depression

First, we calculated the proportion of people in both groups who indicated symptoms of depression and performed univariate analyses to identify potential risk factors associated with symptoms of depression, defined as a total PHQ-9 score > 4. More than 30% of people showed symptoms of depression in both groups (Table 1) Quarantine times, age, marital status, and length of education showed a significant association with symptoms of depression (all *p* ≤ 0.01; Table 3). Multivariate logistic regression confirmed that symptoms of depression were significantly associated with quarantine times [OR 1.371, 95% confidence interval (CI) 1.15–1.635], age (OR 0.644, 95% CI 0.504–0.822), and length of education 1.344(95% CI 1.118–1.615; Table 4).

**Table 3:** Factors associated with symptoms of depression, defined as PHQ-9 score > 4.

**Factor**	**Odds ratio (95% CI)**	***p***
Quarantine times	1.371 (1.149–1.635)	0.01
Sex	0.983 (0.83–1.163)	0.839
Age	0.61 (0.501–0.742)	<0.001
Length of education	1.355 (1.142–1.607)	<0.001
Marital status	0.73 (0.608–0.875)	0.001

*CI, confidence interval; PHQ-9, Patient Health Questionnaire-9*.

**Table 4:** Multivariate logistic regression to identify independent predictors of symptoms of depression, defined as PHQ-9 score > 4.

**Factor**	**OR**	**95% CI**	***p***
Quarantine times	1.371	1.15–1.635	<0.001
Age	0.644	0.504–0.822	<0.001
Length of education	1.344	1.118–1.615	0.002
Marital status	0.912	0.729–1.142	0.423

*CI, confidence interval; OR, odds ratio; PHQ-9, Patient Health Questionnaire-9*.

## Discussion

In this study, we examined the effect of repeated quarantine on the risk of anxiety, depression, and PTSD among individuals in China during the COVID-19 pandemic. Based on a cross-sectional survey, we found that those who had been quarantined twice were more likely to show symptoms of depression than those who have been quarantined only once. There were no significant differences between the two groups of participants with respect to anxiety or PTSD. As far as we know, this is the first study investigating the impact of quarantine times on the psychological and emotional condition of Chinese people during the COVID-19 pandemic.

### Impact of Quarantine Times on Anxiety and PTSD Symptoms

Our findings show that there were no significant differences in the symptoms of anxiety or PTSD between participants who had experienced quarantine once or twice. Anxiety and PTSD are typically associated with long-term stress. For example, extended exposure to stress can significantly increase one's anxiety levels, which may lead to PTSD (17). After implementing several epidemic control measures to fight the COVID-19 pandemic, many cities in Western China relaxed their regulations on blockades, thus reducing the panic experienced by the general population. Many people even reported that the epidemic seemed far away from them (18).

Another reason is that the observed lack of association between quarantine times and symptoms of anxiety or stress in our study may reflect the presence of family support. Chinese people generally have more family support (19). Such support can significantly reduce anxiety and PTSD. Future work should focus on gaining a better understanding of the impact of pandemic prevention measures such as quarantine on the anxiety and PTSD levels, and it should explore factors that can prevent or mitigate the negative effects of such measures.

### Impact of Quarantine Times on Depression Symptoms Such as Hikikomori-Like Social Withdraw

Before the COVID-19, a study which extracted depressive symptom prevalence data from 167 cross-sectional studies and 16 longitudinal studies from 43 countries. The statistics show that the overall pooled crude prevalence of depressive symptoms was 27.2% (20). However, in our study, the prevalence of depressive symptoms was more than 30%. Past studies have shown that both the panic over the epidemic and quarantine can cause the symptoms of depression. This is consistent with our findings (21).

Compared to individuals in our study who had been quarantined only once, those who had been quarantined twice were much more likely to show moderate to severe symptoms of depression. Consistent with our result, a study of Chinese participants reported that home quarantine can lead to social isolation. Social isolation is defined as having few social contacts and little engagement with others and the wider community (22), which can lead to more obvious symptoms of depression (23). This is consistent with the study in other countries (24). During the home quarantine period, people avoid physical contact with family and friends, and they receive groceries and supplies from the community; this means significantly less physical contact with the outside world, which may increase feelings of loneliness (25). Loneliness can be a painful emotional experience that arises from the discrepancy between actual and desired social contact (26).

This phenomenon is very similar to a well-known paradigm, Hikikomori, which was originally described in Japan in the late 20th century and those people avoid social contact for more than 6 months (27). At onset, individuals with hikikomori tend not to suffer and are satisfied because they have escaped real-world stresses. However, longer lasting social isolation gradually increases loneliness (28). According to theories of perceived isolation, the need for social connectedness is deeply ingrained in humans and has evolved along with neural, hormonal, and genetic mechanisms that are directly associated with bonding and companionship. Herd behavior is another crucial factor for survival and reproduction (29). Being social is a human tendency that facilitates interaction: restriction of our movements can lead to psychological distress (22). Thus, as with hikikomori, repeated home quarantine can lead to subjective feelings of loneliness and social isolation. Subjective social isolation and loneliness were found closely related to symptoms of depression and suicidal thoughts, this is a key risk factor for depression and some serious consequences (26, 30).

Therefore, further research on this direction and how we could establish a preventive plan in order to limit the subsequent psychopathological following repeat quarantine are essential. In this regard, we believe that the original hikikomori support programs may serve as an important reference. It developed a family based educational program to reduce the risk of family violence, suicide, and other mental disturbances due to hikikomori, using lectures and role play sessions (31). Generally, home quarantine is based on family units. So, this method is also applicable for them and is a good way to reduce the negative consequences. However, making preventive plans for these problems is not an easy task. In Asian countries, fears are probably deeply rooted in traditional-culture-based shame and social ostracism. Asian countries have higher stigma of mental illness, and patients are more likely to be ostracized by society. Therefore, they are less likely to seek help from psychiatrists or psychologists when dealing with emotional problems such as symptoms of depression (28, 32). As a result, we can increase efforts such as lectures, community training and online educational outreach to raise public awareness about mental illness, which may help reduce stigma and prevent its consequences.

### Repeated Quarantine and Online Gaming Disorder

One study showed that the fear resulting from the COVID-19 disease, and the consequences of home quarantine have been mounting affecting individuals' behaviors. As a result, the prevalence of online gaming disorder among Chinese teenagers is relatively high (33). Isolated populations use the Internet and social media for longer periods due to mobility constraints, which increases the risk of Internet addiction. The study showed that participants experienced severe depression and anxiety during the pandemic and 2.68 and 33.37% of the participants of Chinese adolescents during the pandemic were classified as addicted and possibly addicted to the Internet. The frequency and duration of recreational electronic devices use, the frequency of electronic devices use after 00:00, and the self-score of addiction to electronic products were all significantly higher than those before the epidemic (34). It is consistent with previous findings that stressful emotions such as anxiety and depression may exacerbate pathological Internet use (35).

Reduced outdoor activities, readjusting to online courses after school closures, and poor academic progress led to anxiety and depression, which were exacerbated by repeated home quarantine. Online games are entertaining and easy to access, which may be a common way for children and adolescents to release emotions and stress and escape from reality (36). The Internet, especially online games, can stimulate individuals to have a sense of energy and autonomy and enhance self-esteem. However, Internet addiction, like gambling, is a behavioral addiction, characterized by a progression from pleasure to loss of control and finally to obsession (37). As the times of home quarantine rose, the time teenagers spend playing online games gradually increased. Excessive users will be more focused on Internet and less interested in real life (38).

We are not yet sure of the causal relationship between Online gaming disorder and emotional problems, which suggests that this should be the further research for this direction. Further preventive planning is also needed for the use of the Internet by repeated home quarantine populations. Although free online or telephone counseling was promoted in China during the outbreak, there are still problems such as insufficient counselors and lack of professional assistance. Therefore, the current preventive plan should expand online counseling, and assign fixed online counselors to each class as a unit, and carry out regular psychological lectures and one-to-one psychological counseling.

### Risk Factors Associated With Depression

Age is negatively associated with symptoms of depression. Consistent with previous studies, we found that depression was more prevalent and severe among our participants aged 18–29. A review of 63 studies involving 51,576 participants from numerous countries reported clear associations between loneliness and mental health problems, particularly depression, in children and adolescents during the COVID-19 pandemic (26).

In early 2020, most countries adopted online teaching and suspended in-person classes because of the pandemic. This led many students to isolate at home and alter their sleep habits and lifestyle, which may have increased their stress and challenged their mental health (39). Indeed, loneliness may have a particularly strong impact on young people since they require a social identity and support from peer groups for better mental health. Restricting social opportunities for young people may increase their risk of depression (26).

In our study, subjects' level of education was associated with their psychological status: individuals who had been educated for at least 12 years were more likely to experience depression. This is inconsistent with studies conducted in periods without an epidemic: one study, for example, reported that higher education was associated with better economic and psychological status and therefore lower risk of depression (30). One reason for the association observed in our study is that more educated individuals may have more access to information about the COVID-19 pandemic *via* the Internet and social media, and such exposure can lead to depression and other negative emotions (40). A long-term study of social networks showed that enduring emotional states of depression and happiness can be transmitted through the Internet (41). These emotions would be transmitted to people who constantly receive relevant information. During the pandemic, many people have expressed their fears, worries, and other negative emotions on social media. At the same time, these networks have found it difficult to block false information about COVID-19. This has caused unfounded fears among many Internet users, leading to the development of anxiety and depression (40).

### Effect of Other Demographic Factors

In our study, time spent in quarantine, age, and length of education were associated with symptoms of anxiety, depression, and PTSD. None of the other demographic factors that we measured had a significant effect on mental health in our sample. These results are inconsistent with previous studies. For example, studies conducted in many countries, including China, Turkey, Italy, and Spain, show that quarantine increases symptoms of stress, anxiety, and depression to a greater extent in women than in men (42). One study showed that the gender difference peaked in adolescence but then declined and remained stable in adulthood (43). The participants in this study did not cover adolescents, which may be another reason why the differences were not significant. In addition, we speculate that this inconsistency may be related to the limited nature of the online questionnaire.

### Repeated Quarantine and Post-traumatic Growth Effect

Although the correlation between repeated quarantine and PTSD was not positive in this study, the post traumatic growth effect is a topic worth discussing, and its impact may provide guidance and direction on how to carry out psychological assistance.

Past research has found that not all people exhibit maladaptive responses after having experienced a high-stress life event. Instead, some individuals develop a more positive perspective on life and develop post-traumatic growth (PTG) (44). Positive psychosocial adjustment to a life-changing stressor may counteract the negative effects of adopting health-damaging behaviors due to event-related anxiety (45). Therefore, it is theoretically possible to reduce the risk of predisposing to online gaming disorder and alleviate Hikikomori-like social withdraw during and after the quarantine.

One study, conducted under controlled conditions, found that though it couldn't allay the painful distress symptoms, a 30 min of weekly talking could lead to post-traumatic growth (46). In addition, a research found that post-traumatic growth is more likely when people basic psychological needs are satisfied. previous research has indicated that people' resilience may benefit from a sense of self-efficacy (47), psychological endurance (48), and organization support (49). Besides, a separate point mentioned is age, which is negatively associated with post-traumatic growth, possibly indicating that older people have developed an established adaptation in the past (50) and may respond less well to psychotherapy.

All of these characteristics mentioned above can be used as the current psychological intervention targets for repeated quarantine populations.

### Limitations

Our findings must be considered with caution in the light of certain limitations. First, due to the pandemic and the quarantine situation, we were restricted to using a strategy of online sampling survey to collect data. Since our sample was limited to individuals who used the WeChat platform, cared about their mental health, and were willing to participate in a questionnaire survey, we cannot rule out sampling bias. Indeed, we included only individuals who were >18 and <65 years old. Future work should include a wider range of demographic characteristics, such as household income and size of household population in order to provide a better understanding of the potential risk factors associated with anxiety, depression, and PTSD. Second, since this is a cross-sectional study, our analysis could not capture potential changes in the variables that we measured, or in their relationships with symptoms of depression, anxiety or stress. Future research should longitudinally examine the effects of quarantine on mental well-being, and the sample should also include children, adolescents, and the elderly. Third, our research cannot explain why family support in home quarantine reduce anxiety but not depression. This is also a limitation of our research.

## Conclusion

The results of this study show that increasing the times of quarantine during the COVID-19 pandemic can increase the prevalence and severity of depression, without affecting anxiety or PTSD-related symptoms. Therefore, public health officials and clinicians should focus more on developing effective interventions for individuals with depression, especially in areas affected by epidemics.

## Data Availability Statement

The raw data supporting the conclusions of this article will be made available by the authors, without undue reservation.

## Author Contributions

QT was a major contributor in writing the manuscript. YW mainly responsible for data collection. JL provided guidance to the study design. DL mainly responsible for data analysis. JX and XH mainly responsible for directing and revising the writing of manuscript. All authors read and approved the final manuscript.

## Funding

This study was funded by Sichuan Provincial Science and Technology Support Program (Grant ID: 2019YFS0218) and (Grant ID: 2020YFS0587).

## Conflict of Interest

The authors declare that the research was conducted in the absence of any commercial or financial relationships that could be construed as a potential conflict of interest.

## Publisher's Note

All claims expressed in this article are solely those of the authors and do not necessarily represent those of their affiliated organizations, or those of the publisher, the editors and the reviewers. Any product that may be evaluated in this article, or claim that may be made by its manufacturer, is not guaranteed or endorsed by the publisher.
